# Case Report: Fetomaternal hemorrhage and its association with pronounced neonatal anemia

**DOI:** 10.3389/fped.2024.1423786

**Published:** 2024-10-08

**Authors:** Peng Li, Hua Shu, Peng Lin, Jishui Wang, Di Zhang, Dongmei Man, Fengge Wang

**Affiliations:** ^1^ College of Clinical Medicine, Jining Medical University, Jining, Shandong, China; ^2^ Department of Obstetrics, Affiliated Hospital of Jining Medical University, Jining Medical University, Jining, Shandong, China

**Keywords:** fetomaternal hemorrhage, severe neonatal anemia, pregnancy, obstetrics, case report

## Abstract

Fetomaternal hemorrhage (FMH) is a perplexing obstetric condition that predominantly occurs during the third trimester or at the time of delivery. Its insidious and non-specific onset often leads to diagnostic challenges. The underlying pathophysiology of FMH remains incompletely understood, though it is primarily attributed to compromise of the placental barrier. The severity of the condition is intrinsically associated with the volumn of blood loss, the hemorrhage rate, and the presence of alloimmunity. Upon the occurrence of severe FMH, it can rapidly lead to intrauterine distress, fetal anemia, and the possibility of fetal demise, presenting a considerable threat to both maternal and neonatal well-being. In this article, I present a substantial case of FMH and conduct a systematic review of the current scientific literature regarding the etiology, clinical manifestations, diagnostic approaches, treatment highlights, and prognosis of this condition. The objective of this work is to improve clinicians’ comprehension and diagnostic proficiency concerning FMH.

## Introduction

Fetomaternal hemorrhage (FMH), first described by Weiner (1) in 1948, refers to a clinical syndrome in which a defined volume of fetal blood enters the maternal circulation via a disrupted placental barrier during pregnancy or childbirth, leading to diverse levels of fetal blood loss or maternal hemolytic reactions. *in situ*ations where the maternal and fetal blood types are incompatible, there exists a risk of a hemolytic transfusion reaction occurring between the two circulatory systems (1). Minor bidirectional transfusions may transpire throughout any phase of typical pregnancy (2). Typically, more than 99% of cases of maternal-fetal transfusions involve blood loss of less than 15 milliliters; however, when the blood loss reaches 25–30 ml (which accounts for 20% of the fetal placental blood volume), or in the event of acute hemorrhage, it can lead to severe complications including fetal anemia, edema, and even intrauterine death (3). The incidence of FMH varies according to the volume of bleeding (3). The incidence of FMH in pregnant women is 3 per 1,000 with a blood loss of 30 ml or more, 1 per 1,000 with a blood loss of 80 ml or more, and ranges from 1 per 2,800 to 1 per 3,000 when the blood loss exceeds 150 ml (4–6). Presently, FMH is primarily documented as isolated cases, indicating a deficiency in clinician awareness regarding this medical condition. *in situ*ations involving unexplained fetal anemia or intrauterine fetal death, it is prudent to consider the execution of FMH testing to more accurately identify the underlying etiology.

## Case data

A 36-year-old woman, para 1 and gravida 4, with blood type O Rh positive, was referred to our hospital at 36 weeks and 4 days of gestation due to a reported decrease in fetal movements over the past three days. She had no documented history of abdominal trauma during her pregnancy. Prior to this admission, she was diagnosed with gestational diabetes mellitus and undifferentiated connective tissue disease. Prenatal screening tests yielded normal results, and her screening for Group B streptococcus as well as serologies for congenital infections returned negative. Upon admission, her physical examination was largely unremarkable, showing no uterine contractions, stable blood pressure and heart rate, and a long, closed cervix without signs of vaginal bleeding. Ultrasound findings at admission indicated a singleton fetus with growth measurements consistent with 37 weeks gestation, devoid of fetal anomalies or hydramnios, and the placenta exhibited normal morphology and positioning. However, cardiotocography (CTG) revealed significantly reduced baseline variability. In response to fetal intrauterine distress, we executed an urgent cesarean section, resulting in the delivery of a markedly pale female infant. During the operative procedure, the amniotic fluid was found to be contaminated with meconium, while both the placenta and umbilical cord appeared normal. The newborn exhibited pale skin, edema, cyanotic lips, an inability to cry, absence of spontaneous respiration, and a heart rate of 40 beats per minute. Apgar scores recorded at 1, 5, and 10 min postpartum were 1, 5, and 6, respectively. The placental blood gas analysis revealed: pH = 6.88, Pco2 = 108 mmHg, BE = −13.0 mmol/L, and hemoglobin concentration of 32 g/L, indicating decompensated acidosis, which was addressed with alkaline fluid administration. Following resuscitation measures for asphyxia, the neonate was transferred to the NICU for management due to “neonatal asphyxia (severe), neonatal anemia (extremely severe), and neonatal respiratory failure in a preterm infant”. Both the neonatal and maternal blood types were confirmed as RhD type O, and the infant's Coombs test was negative. The complete blood count for the newborn reveals a white blood cell count of 29.76 × 10^9/L, a red blood cell count of 1.09 × 10^12/L, a hemoglobin level of 41 g/L, a hematocrit of 13.5%, a platelet count of 208 × 10^9/L, a neutrophil count of 16.5 × 10^9/L, and reticulocyte count at 556.8 × 10^9/L. The reduction in hemoglobin and elevation in reticulocyte count suggest that the newborn experienced blood loss a few days prior, resulting in severe anemia (7).We hypothesize that the observed blood loss occurred a few days before the cesarean section and may be correlated with a decrease in fetal movements over the past three days. The chest ultrasound of the newborn indicates lung consolidation, neonatal pneumonia, and right pleural effusion, which correlate with the elevated white blood cell and neutrophil counts.The neonate received normal saline for volume expansion and dopamine to enhance circulation. Blood transfusions were administered at 15 ml/kg for three sessions, totaling 120 ml, resulting in significant improvement of severe anemia. (a post-transfusion hemoglobin level of 175 g/L). Subsequent management focused on active infection control, correction of hypoalbuminemia, and maintenance of fluid and electrolyte balance, which progressively stabilized the neonate's condition, leading to discharge after an 11-day hospitalization.

## Discussion

The etiology of FMH is complex and associated with numerous obstetric factors, including fetal conditions (such as anomalies, multiple gestations, and intrauterine fetal demise), placental abnormalities (including placenta previa, placental abruption, tumors, and umbilical vein thrombosis), maternal trauma, and obstetric interventions (like amniocentesis) (7). Previous diagnoses of gestational diabetes and undifferentiated connective tissue disease may increase the risk of placental barrier damage; however, clinically, over 80% of cases of FMH with hemorrhage volumes exceeding 30 ml remain idiopathic (8). Massive FMH is a rare occurrence that is primarily documented by case reports. No established risk factors associated with massive FMH have been identified (9). It is often diagnosed in contexts such as severe neonatal anemia, unexplained stillbirth, or non-immune fetal hydrops (10).

**Figure 1:**
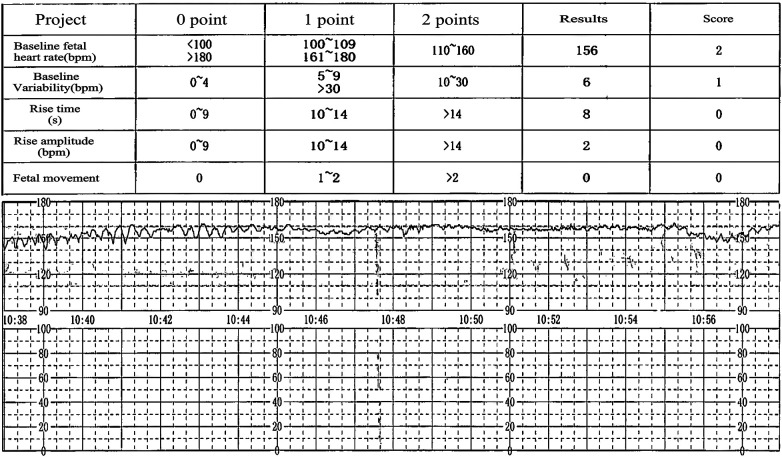
CTG monitoring conducted upon admission.

The severity of FMH is related to the rate and volume of fetal blood loss (11). Clinical manifestations associated with FMH include neonatal anemia, stillbirth, preterm labor, intrauterine growth restriction, fetal hydrops, reduced fetal movement, and abnormal cardiotocography(CTG) patterns (12). Most patients with FMH initially exhibit reduced fetal movements, accompanied by abnormal CTG patterns, such as bradycardia or tachycardia, diminished variability, and sinusoidal or late deceleration, marking a critical juncture for the identification of FMH (13). A decline or absence of fetal movement, sinusoidal fetal heart rate pattern, and fetal edema constitute the classic triad indicative of massive FMH (14), aligning with the clinical manifestations observed in the patient discussed herein (Figure 1 & Supplementary Figure S1). A decline in fetal movements may serve as an early indicator of FMH, whereas fetal edema indicates a chronic condition stemming from FMH (7).

Based on the Apgar scores and the placental blood gas analysis of the neonate, we determine that the infant is suffering from severe neonatal asphyxia, profound neonatal anemia, and neonatal respiratory failure. It's imperative to recognize that the neonate necessitates not only effective ventilation but also an urgent blood transfusion at this juncture. The identification of meconium-stained amniotic fluid indicates intrauterine hypoxia, which has precipitated acidosis in the neonate. This acidosis stimulates the respiratory center due to increased carbon dioxide levels, potentially leading to significant aspiration of amniotic fluid. Furthermore, substantial blood loss in the neonate poses a risk of hypovolemic shock. These factors are likely critical contributors to the asphyxia observed. According to Clinical Anesthesiology, neonates and preterm infants with a hematocrit lower than 30% are at a higher risk for apnea; thus, it is advised to maintain a hematocrit above 35% for infants younger than three months (15). The infant's hemoglobin level is measured at 41 g/L, with a hematocrit of 13.5%, markedly below the survivable threshold. Therefore, I infer that the neonate has developed a massive FMH, which may elevate the risk of preterm delivery.

The clinical diagnosis of FMH primarily relies on the prompt responses of physicians and auxiliary examinations (6). Common supplementary tests include the Rosettle screen, Kleihauer-Betke test, and flow cytometry, all of which have been approved by the U.S. Food and Drug Administration (16). However, these supplementary tests are not routinely incorporated into standard screenings in many regions, highlighting a significant lack of awareness regarding FMH (6). Furthermore, measurements of fetal hemoglobin and the peak systolic velocity of the middle cerebral artery can provide crucial indications, as these metrics may signal fetal anemia (17, 18). Notably, placentas with FMH often exhibit distinctive characteristics, such as parenchymal pallor, an elevated count of nucleated red blood cells, and the presence of syncytial knots, alongside the expression of vascular endothelial growth factor (VEGF), CD34, and CD31 in capillary endothelial cells (19). Instances of concurrent intraplacental choriocarcinoma and FMH have been documented, prompting placental pathology examinations in FMH cases when feasible to enhance the detection of potential intraplacental choriocarcinoma (20).

The complications associated with FMH are numerous, including fetal distress, intrauterine growth restriction, postnatal respiratory failure, central nervous system dysfunction, persistent pulmonary hypertension, disseminated intravascular coagulation, pulmonary hemorrhage, and renal failure (8). The prognosis for infants affected by FMH is generally poor, with mortality rates ranging from 8% to 38% (21). Research indicates that the incidence of neurological injuries in FMH is between 4% and 18%, manifesting primarily as intraventricular hemorrhage, cerebral infarction, ventricular enlargement, periventricular white matter softening, cerebral atrophy, and cerebral palsy (22). The hemoglobin levels in the fetus prior to treatment can partially reflect the prognosis of FMH; specifically, lower hemoglobin levels correlate with a worse outcome (23).

Upon receiving a confirmed diagnosis of fetal-maternal hemorrhage (FMH), an early and tailored treatment protocol should be implemented, taking into account gestational age and disease severity.

A. For gestational age ≤ 32 weeks: In cases of mild FMH and slight anemia, observation may continue if the cardiotocography (CTG) and biophysical profile results are within normal limits. For patients at elevated risk for preterm labor, administration of glucocorticoids and magnesium sulfate is advised to enhance fetal pulmonary and neurological development. In cases of severe FMH, intrauterine transfusion (IUT) should be considered while remaining vigilant for the potential recurrence of FMH (24).
B. For gestational age between 32 weeks and 36 weeks: A proficient multidisciplinary team is essential to evaluate the risks associated with IUT and premature delivery (25).
C. For gestational age > 36 weeks: Prompt delivery should be pursued, alongside personalized blood transfusion strategies based on the degree of anemia present in the neonate, with the objective of minimizing maternal and fetal adverse outcomes (26).

Regrettably, we overlooked conducting the FMH testing and placental pathology examination, which necessitates a thorough reflection based on the existing pertinent literature. Although the occurrence of FMH is relatively infrequent, its consequences can be profoundly detrimental for both the mother and the fetus (27). Obstetricians must remain vigilant regarding reduced fetal movements and abnormal cardiotocography (CTG) trace, particularly during the third trimester of pregnancy (28). It is essential to conduct FMH testing promptly when confronted with unexplained anemia and edema.

## Data Availability

The original contributions presented in the study are included in the article/Supplementary Material, further inquiries can be directed to the corresponding authors.

## Supplementary material

Supplementary Figure S1 Pre-admission CTG tracing.
