# Supplementary figures and images for: Case Report: Fetomaternal hemorrhage and its association with pronounced neonatal anemia
Source: Front Pediatr. 2024 Oct 8;12:1423786. doi: 10.3389/fped.2024.1423786 (PMC11497261; doi:10.3389/fped.2024.1423786)

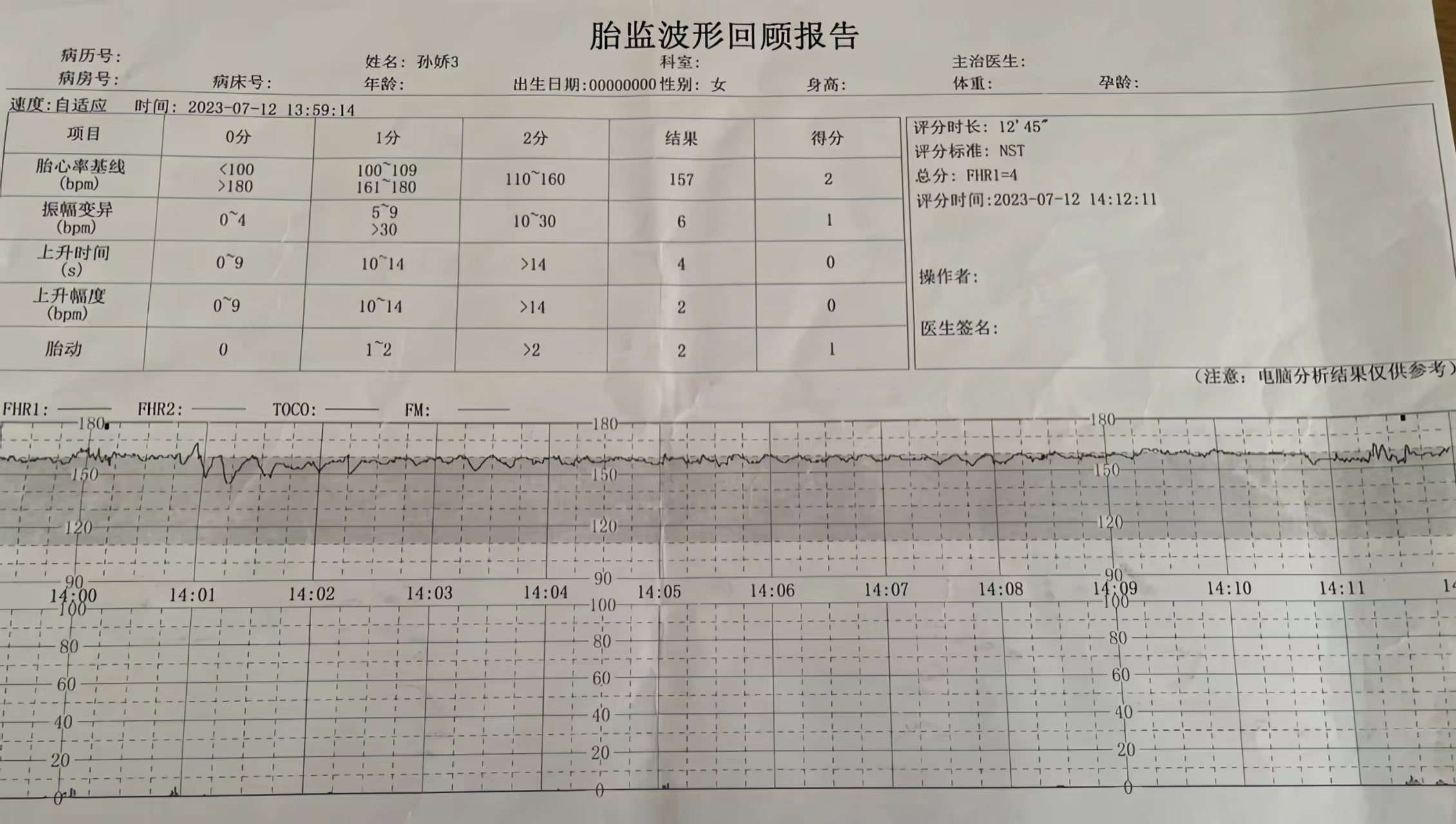

Supplement: Supplementary Figure S1 — Pre-admission CTG tracing. [file Image1.jpeg]
